# Clinical relevance of inflammatory markers in the evaluation of severity of ulcerative colitis: A retrospective study

**DOI:** 10.1515/biol-2025-1088

**Published:** 2025-05-05

**Authors:** Tao He, Lian-Qiang Song, Xiao-Yu Weng, Peng Pan, Hui Ding, Mei-Qin Liu, Shi-Lin Qiu, Shan-Ming Sun

**Affiliations:** Department of Gastroenterology, Weifang People’s Hospital, Shandong Second Medical University, No. 151 of Guangwen Road, Kuiwen District, Weifang, 261000, China; Department of Endocrinology, Weifang Respiratory Disease Hospital, Shandong Second Medical University, Weifang, 261000, China

**Keywords:** disease assessment, neutrophil/lymphocyte ratio, platelet/lymphocyte ratio, ulcerative colitis

## Abstract

This study aimed to investigate the clinical relevance of inflammatory markers in the severity assessment of ulcerative colitis (UC). The inflammatory markers included the neutrophil-to-lymphocyte ratio (NLR), platelet-to-lymphocyte ratio (PLR), C-reactive protein (CRP), erythrocyte sedimentation rate (ESR), and calcium ion (Ca^2+^) levels. A retrospective analysis was on 110 patients with UC and 52 patients with irritable bowel syndrome (IBS), admitted to Weifang People’s Hospital between June 2019 and February 2021. UC severity was classified using the modified Mayo score and the Montreal classification system. The study assessed the predictive accuracy and correlation of these inflammatory markers with UC severity and extent. Levels of NLR, PLR, CRP, ESR, and Ca^2+^ were significantly elevated in individuals with UC compared to those with IBS. Among patients with UC, significant differences in these markers were observed across varying severity levels as defined by the modified Mayo score. However, aside from ESR, no significant differences were noted in NLR, PLR, CRP, or Ca^2+^ levels across groups defined by lesion extent. Receiver operating characteristic curve analysis indicated that NLR exhibited the highest predictive accuracy for UC, with a cut-off value of 2.603 yielding a sensitivity of 0.545, specificity of 0.288, and an area under the curve (AUC) of 0.896. The combined use of NLR, PLR, CRP, ESR, and Ca^2+^ demonstrated superior predictive performance, achieving an AUC of 0.972, sensitivity of 0.927, and specificity of 0.923 at a cut-off value of 0.455. NLR, PLR, CRP, ESR, and Ca^2+^ exhibit predictive value for UC, with NLR demonstrating the highest individual predictive performance. The combined use of these markers enhances predictive accuracy, highlighting their potential application in clinical practice for the evaluation of severity UC. Due to ethical considerations at our institution, the IBS group was used as a substitute for healthy controls. The IBS group was included solely for the calibration and testing of inflammatory biomarkers, as well as for subsequent analysis of their role in assessing UC severity.

## 1 Introduction

Ulcerative colitis (UC) is a chronic, nonspecific inflammatory bowel disease (IBD) characterized by persistent or recurrent episodes of diarrhea, bloody stools, purulent mucus, abdominal pain, urgency, and varying degrees of systemic symptoms [1]. Despite extensive research, the underlying pathogenesis of UC remains unclear. The disease has become a significant global health concern, with its incidence rising rapidly in China [2,3].

Colonoscopy and mucosal biopsy are considered the gold standards for diagnosing UC and evaluating its severity. However, these methods are invasive, costly, and associated with potential complications such as bleeding and perforation, which often result in low patient compliance. This underscores the need for simpler, safer, and more cost-effective serological markers to assess UC severity. Despite their availability, currently used serological markers for the severity assessment of UC have demonstrated suboptimal accuracy [4].

In recent years, the neutrophil-to-lymphocyte ratio (NLR) and platelet-to-lymphocyte ratio (PLR) have gained recognition as novel inflammatory markers, with studies highlighting their relevance to infections, tumors, and autoimmune diseases [5–8]. Additionally, hypocalcemia is a common complication in UC, with evidence suggesting a correlation between calcium ion (Ca^2+^) levels and disease severity [9].

The present study aims to evaluate the predictive value of NLR, PLR, C-reactive protein (CRP), erythrocyte sedimentation rate (ESR), and Ca^2+^ levels, both individually and in combination, for UC. Furthermore, this study seeks to assess their utility in determining disease severity, providing insights to support clinical monitoring and the evaluation of treatment outcomes in UC.

## 2 Data and methods

### 2.1 General data

This study included 110 individuals diagnosed with UC and 52 individuals diagnosed with irritable bowel syndrome (IBS) who were hospitalized at Weifang People’s Hospital between June 2019 and February 2021. Patients with IBS served as the control group, as IBS is widely regarded as a functional gastrointestinal disorder, and current evidence suggests no statistically significant differences in serological markers between patients with IBS and healthy individuals [10].

The UC group comprised 48 males and 62 females, with a mean age of 45.7 ± 16.9 years, while the control group included 20 males and 32 females, with a mean age of 38.2 ± 12.6 years. A comparison of demographic data between the two groups indicated no statistically significant differences (*p* > 0.05), ensuring their comparability. The study protocol was approved by the Medical Ethics Committee of Weifang People’s Hospital (approval number: wfj-20220806).


**Informed consent:** Informed consent has been obtained from all individuals included in this study.
**Ethical approval:** The research related to human use has been complied with all the relevant national regulations, institutional policies and in accordance with the tenets of the Helsinki Declaration, and has been approved by the Ethics Committee of Weifang people’s Hospital (Approval number is KYLL20240327-1).

### 2.2 Inclusion and exclusion criteria

Inclusion criteria: UC diagnosis was confirmed based on the “Consensus Opinions on the Diagnosis and Treatment of Inflammatory Bowel Disease” issued by the Inflammatory Bowel Disease Group of the Chinese Society of Gastroenterology, Chinese Medical Association, 2023 [1]. IBS diagnosis was established using the Rome IV criteria [11].

Exclusion criteria: (1) individuals younger than 16 years or older than 85 years; (2) individuals with concurrent conditions, including diabetes, coronary artery atherosclerotic heart disease, rheumatic diseases, acute or chronic infectious diseases, malignancies, or renal dysfunction; (3) individuals with incomplete clinical data; and (4) pregnant or lactating women.

### 2.3 Research methods

The following clinical and laboratory data were collected for each participant: name, age, gender, stool characteristics, results of the first colonoscopy performed after admission, CRP, ESR, complete blood count, and electrolytes. The NLR and PLR were calculated using the equations: NLR = neutrophil count/lymphocyte count; PLR = platelet count/lymphocyte count. Comparisons of the five serological indicators (NLR, PLR, CRP, ESR, and Ca^2+^) were conducted between patients with UC and IBS, across different extents of UC lesions, and among varying severities of UC. Variations in NLR, PLR, CRP, ESR, and Ca^2+^ levels were analyzed in relation to the extent of UC lesions, and their correlation with the modified Mayo score was assessed.

The predictive utility of NLR, PLR, CRP, ESR, and Ca^2+^, both individually and in combination, was evaluated using receiver operating characteristic (ROC) curve analysis.

The severity of UC was determined using the modified Mayo score [12], which consists of four components: bowel movement frequency, rectal bleeding, endoscopic findings, and global assessment by the physician. Each component was scored on a scale of 0–3, with a total score categorized as follows: ≤2 with no individual component >1: symptom remission; 3–5: mild disease; 6–10: moderate disease; and 11–12: severe disease.

The extent of UC was classified based on the Montreal classification including pan-colitis [13]:

(1) Proctitis (E1): disease confined to the rectum; (2) left-sided colitis (E2): disease extending from the rectum to the splenic flexure; (3) extensive colitis (E3): disease extending proximal to the splenic flexure.

Endoscopic disease activity, assessed using white-light endoscopy, was graded according to the Mayo Endoscopic Score (MES) [14]: 0: inactive disease; 1: mild disease; 2: moderate disease; 3: severe disease.

### 2.4 Sample collection and laboratory analysis

Fasting blood samples were collected from the median cubital vein on the second day following admission. The following analyses were conducted: complete blood count: performed using EDTA-K2 anticoagulant tubes with an automated hematology analyzer; CRP and calcium ion (Ca^2+^) levels: measured from serum samples collected in gel separator tubes using a biochemical analyzer; and ESR: assessed using blood samples collected in sodium citrate tubes with an automated ESR analyzer.

### 2.5 Fecal calprotectin (FC) assessment

Stool samples were collected within 3 days of undergoing sigmoidoscopy. Samples were processed using the ‘Faecal Sample Preparation Kit’ (Roche Diagnostics). FC levels were measured using two methods: “Quantum Blue” assay (Buhlmann, Switzerland) and the EliA fluoroimmunoassay (Phadia, ThermoFisher, Sweden).

### 2.6 Statistical methods

Data analysis was performed using SPSS 25.0 software. For continuous variables following a normal distribution, results were expressed as mean ± standard deviation (Mean ± SD), and comparisons between groups were conducted using the independent samples *t*-test. For variables that did not follow a normal distribution, data were presented as the median and interquartile range, and differences between groups were assessed using the Mann–Whitney *U* test or the Kruskal–Wallis rank-sum test.

Logistic regression analysis was utilized to identify predictive factors for UC. Among the inflammatory markers, NLR, PLR, CRP, ESR, and Ca^2+^ were deemed for inclusion in the regression model based on their statistical significance in univariate analysis.

The predictive reliability of the markers was evaluated using ROC curves. Specificity and sensitivity values were calculated for each indicator, and the area under the curve (AUC) was used as a measure of predictive performance. The AUC ranges from 0.5 to 1, with higher values indicating greater predictive reliability. An AUC of 0.5 reflects no predictive ability, while values closer to 1 indicate strong predictive performance. A *p*-value of less than 0.05 was considered statistically significant for all analyses.

## 3 Results

### 3.1 Participant demographics and clinical data

A total of 110 patients with UC and 52 patients with IBS who met the inclusion criteria were included in this study. Among the UC group, 48 patients were male (43.6%, 48/110) and 62 were female (56.4%, 62/110), with an age range of 21–69 years and a mean age of 45.7 ± 16.9 years. The average disease duration for patients with UC was 6 years, ranging from 2 to 15 years. In the IBS group, 20 patients were male (38.5%, 20/52) and 32 were female (61.5%, 32/52), with an age range of 24–65 years and a mean age of 38.2 ± 12.6 years. Statistical analysis showed no significant differences in sex, age, or other demographic characteristics between the UC and IBS groups (*p* > 0.05). Further details are provided in Table 1.

**Table 1:** Basic data

Group	Cases	Gender (male/female)	Age (years, \[\bar{X}]\] ± *S*)	Duration of illness (years, IQR)
UC group	110	48/62	45.7 ± 16.9	6 (2, 15)
IBS group	52	20/32	38.2 ± 12.6	—

Abbreviations: IBS, irritable bowel syndrome; UC, ulcerative colitis.

### 3.2 Comparison of NLR, PLR, CRP, ESR, and Ca^2+^ between the UC and IBS groups

Patients in the UC group exhibited significantly higher levels of NLR, PLR, CRP, and ESR compared to the IBS group. In contrast, the UC group demonstrated significantly lower levels of Ca^2+^. These differences were statistically significant (*p* < 0.05) and are summarized in Table 2.

**Table 2:** Comparing NLR, PLR, CRP, ESR, and Ca^2+^ levels between the UC and IBS groups

Group	UC group	IBS group	*Z* value	*p* value
NLR	3.84 (3.07–4.50)	1.94 (1.72–2.88)	−8.126	<0.001
PLR	161.99 (134.05–201.82)	138.28 (103.87–153.33)	−4.768	<0.001
CRP	18.00 (12.14–23.94)	8.33 (6.39–11.00)	−7.545	<0.001
ESR	28.50 (19.75–38.50)	10.53 (5.7–19.13)	−6.801	<0.001
Ca^2+^	2.22 (2.09–2.37)	2.35 (2.27–2.49)	−4.682	<0.001

Abbreviations: IBS, irritable bowel syndrome; UC, ulcerative colitis; NLR, neutrophils–lymphocytes ratio; PLR, platelet–lymphocytes ratio; CRP, C-reactive protein; ESR, erythrocyte sedimentation rate; Ca^2+^, calcium ion.

### 3.3 Comparison of NLR, PLR, CRP, ESR, and Ca^2+^ across different disease severities in the UC group

Among the 110 patients with UC, 17 cases were classified as mild, 72 as moderate, and 21 as severe. Serological markers, including NLR, PLR, CRP, and ESR, exhibited an upward trend with increasing disease severity, progressing from mild to moderate to severe UC. Conversely, Ca^2+^ levels showed a significant downward trend as UC severity increased. These variations in NLR, PLR, CRP, ESR, and Ca^2+^ levels among patients with differing disease severities were statistically significant (*p* < 0.05), as detailed in Table 3.

**Table 3:** Comparing NLR, PLR, CRP, ESR, and Ca^2+^ levels among patients with UC with different disease severities

Group	Mild (*n* = 17)	Moderate (*n* = 72)	Severe (*n* = 21)	*p* value
NLR	2.91 (2.57–3.63)	3.82 (3.17–4.32)	4.93 (3.97–5.85)	<0.001
PLR	127.27 (100.62–146.55)	165.43 (147.16–191.54)	218.54 (154.22–270.35)	<0.001
CRP	14.29 (9.60–18.32)	18.15 (13.46–23.31)	18.55 (12.19–32.62)	0.029
ESR	23.00 (12.00–32.50)	29.00 (19.50–42.00)	34.00 (24.00–42.50)	0.040
Ca^2+^	2.37 (2.24–2.47)	2.20 (2.10–2.37)	2.13 (2.07–2.33)	0.005

Abbreviations: NLR, neutrophils–lymphocytes ratio; PLR, platelet–lymphocytes ratio; CRP, C-reactive protein; ESR, erythrocyte sedimentation rate; Ca^2+^, calcium ion.

### 3.4 Comparison of NLR, PLR, CRP, ESR, and Ca^2+^ levels across Montreal classification subgroups in the UC group

Among the 110 patients with UC, lesion extent was classified according to the Montreal classification: 26 cases were categorized as E1, 40 as E2, and 44 as E3. ESR levels demonstrated a statistically significant upward trend with increasing lesion extent from E1 to E3 (*p* < 0.05). Although NLR and PLR levels exhibited an increasing trend, and Ca^2+^ levels showed a decreasing trend with greater lesion extent, these differences were not statistically significant (*p* > 0.05). Similarly, CRP levels were highest in patients with E2-type lesions; however, no statistically significant correlation was observed between CRP levels and lesion extent (*p* > 0.05). Further details are presented in Table 4.

**Table 4:** Comparison of NLR, PLR, CRP, ESR, and Ca^2+^ levels in patients with UC having different lesion ranges

Group	E1 (*n* = 26)	E2 (*n* = 40)	E3 (*n* = 44)	*p* value
NLR	3.58 (2.89–4.39)	3.93 (3.18–4.31)	3.99 (3.13–3.99)	0.169
PLR	156.30 (133.23–181.68)	160.79 (136.51192.95)	168.65 (121.71–213.87)	0.720
CRP	17.60 (12.78–21.15)	18.98 (12.30–26.25)	17.98 (11.68–23.20)	0.592
ESR	12.00 (9.00–14.50)	29.00 (23.00–34.75)	38.50 (29.00–53.75)	<0.001
Ca^2+^	2.30 (2.15–2.41)	2.21 (2.11–2,37)	2.19 (2.09–2.37)	0.498

Abbreviations: NLR, neutrophils–lymphocytes ratio; PLR, platelet–lymphocytes ratio; CRP, C-reactive protein; ESR, erythrocyte sedimentation rate; Ca^2+^, calcium ion.

### 3.5 Comparison of inflammatory markers based on MES in the UC group

Among the 110 patients with UC, 11 cases were classified as MES ≤ 1, 65 as MES = 2, and 34 as MES = 3. NLR, PLR, and FC levels demonstrated an upward trend with increasing MES scores (from MES ≤ 1 to MES = 2 and MES = 3), while Ca^2+^ levels exhibited a corresponding downward trend. These differences were statistically significant (*p* < 0.05). In contrast, CRP and ESR levels showed no significant correlation with MES scores, and the differences were not statistically significant (*p* > 0.05). Further details are presented in Table 5.

**Table 5:** Logistic analysis of NLR, PLR, CRP, ESR, and Ca^2+^ in prediction of UC

Variable	*B* value	Standard error	Wald value	*p* value	95% CI
NLR	−1.494	0.409	13.366	<0.001	0.101–0.500
PLR	−0.004	0.012	0.103	0.748	0.974–1.019
CRP	−0.309	0.084	13.385	<0.001	0.623–0.867
ESR	−0.091	0.030	9.089	0.003	0.860–0.969
Ca^2+^	3.462	2.478	1.951	0.163	0.009–2.585

Abbreviations: NLR, neutrophils–lymphocytes ratio; PLR, platelet–lymphocytes ratio; CRP, C-reactive protein; ESR, erythrocyte sedimentation rate; Ca^2+^, calcium ion.

### 3.6 Logistic regression analysis of inflammatory markers and predictive factors of UC

Logistic regression analysis was performed on serum markers that exhibited statistically significant differences between the UC and IBS groups: NLR, PLR, CRP, ESR, and Ca^2+^. The results indicated that NLR, CRP, and ESR are predictive factors for the UC. Detailed findings are summarized in Table 6.

**Table 6:** Efficacy of NLR, PLR, CRP, ESR, and Ca^2+^ in predicting UC independently and jointly

Index	AUC	Cut-off value	Youden’s index	Sensitivity	Specificity
NLR	0.896	2.60	0.657	0.945	0.712
PLR	0.732	148.93	0.394	0.664	0.731
CRP	0.868	14.22	0.691	0.691	1.000
ESR	0.831	20.84	0.553	0.745	0.808
Ca^2+^	0.728	−2.23	0.450	0.527	0.923
Jointly	0.972	−0.46	0.850	0.927	0.923

Abbreviations: NLR, neutrophils–lymphocytes ratio; PLR, platelet–lymphocytes ratio; CRP, C-reactive protein; ESR, erythrocyte sedimentation rate; Ca^2+^, calcium ion.

### 3.7 ROC analysis of inflammatory biomarkers for UC prediction

The predictive value of NLR, PLR, CRP, ESR, and Ca^2+^ in UC was evaluated using ROC curve analysis. Among the five biomarkers, NLR demonstrated the highest sensitivity, whereas CRP exhibited the highest specificity. NLR emerged as the most effective individual marker for UC prediction, with an optimal cut-off value of 2.603, yielding a sensitivity of 0.555, a specificity of 0.288, and an AUC of 0.896.

**Figure 1:**
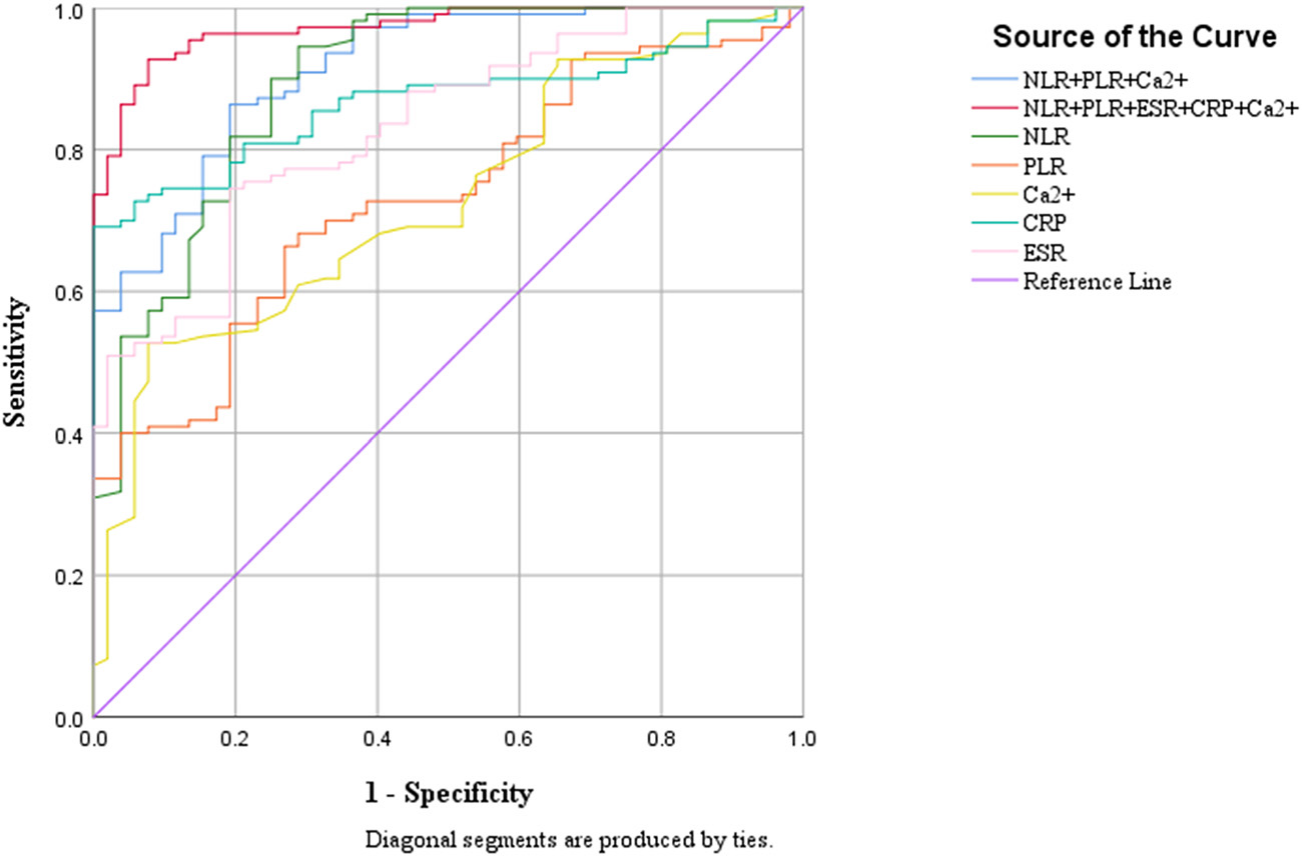
ROC curves for inflammatory markers in the diagnosis of UC, presented both individually and in combination (reference line).

The combined use of all five biomarkers (NLR, PLR, CRP, ESR, and Ca^2+^) significantly enhanced predictive performance. At a cut-off value of 0.455, the combined model achieved a sensitivity of 0.927, a specificity of 0.923, and an AUC of 0.972, surpassing the efficacy of individual markers. Additional details are provided in Table 7 and illustrated in Figure 1.

**Table 7:** Evaluation of UC using inflammatory markers and their combinations

Index	AUC	Accuracy	Sensitivity	Specificity	Cut-off value
NLR	0.896	0.430	0.555	0.288	2.603
PLR	0.732	0.315	0.336	0.269	148.933
CRP	0.868	0.210	0.309	1.000	14.222
ESR	0.831	0.235	0.255	0.192	20.843
Ca^2+^	0.728	0.654	0.527	0.923	2.225
NLR + PLR + Ca^2+^	0.914	0.846	0.864	0.808	0.397
NLR + PLR + Ca^2+^ + ESR + CRP	0.972	0.926	0.927	0.923	0.455

Abbreviations: NLR, neutrophils–lymphocytes ratio; PLR, platelet–lymphocytes ratio; CRP, C-reactive protein; ESR, erythrocyte sedimentation rate; Ca^2+^, calcium ion.

## 4 Discussion

UC is a chronic, nonspecific IBD characterized by alternating periods of relapse and remission. It follows a protracted clinical course and is associated with an increased risk of colorectal carcinogenesis. Early diagnosis and continuous monitoring of disease progression are essential for optimizing therapeutic strategies and improving prognosis.

Currently, UC diagnosis relies on a combination of clinical symptoms, hematological findings, colonoscopy, and mucosal biopsy. While colonoscopy and mucosal biopsy remain the gold standards for diagnosing UC and evaluating disease severity, these procedures are invasive, costly, and associated with patient discomfort. Such factors often result in poor compliance, adversely impacting disease management.

In recent years, novel biomarkers, such as FC and anti-neutrophil cytoplasmic antibodies, have been incorporated into clinical practice for UC assessment [15]. Despite their potential, these biomarkers are limited by low specificity, high costs, and the need for specialized testing, which constrain their widespread application. Consequently, there remains a pressing need for diagnostic tools that are highly specific, cost-effective, and practical, to facilitate UC diagnosis and guide clinical treatment effectively.

The findings of this study demonstrate that NLR, PLR, CRP, ESR, and Ca^2+^ levels are significantly elevated in patients with UC compared to those with IBS, supporting their utility as differential predictive markers. Statistically significant differences in NLR, PLR, CRP, ESR, and Ca^2+^ levels were observed across varying degrees of disease activity in patients with UC. Among these markers, ESR levels showed a significant association with lesion extent, while NLR, PLR, CRP, and Ca^2+^ levels did not reveal significant differences in assessing lesion extent. Logistic regression analysis identified NLR, CRP, and ESR as predictive factors for UC.

Regarding predictive efficacy, ROC curve analysis demonstrated that NLR exhibited the highest predictive performance among the five markers. A cut-off value of 2.603 for NLR yielded a sensitivity of 0.555, a specificity of 0.288, and an AUC of 0.896. These findings are consistent with those reported by Akpinar et al., who observed significantly higher NLR levels in patients with endoscopic active UC compared to those in remission [16]. Furthermore, their study demonstrated a positive correlation between NLR and the Rachmilewitz endoscopic activity index, emphasizing NLR’s potential as a predictive marker for endoscopic disease activity during colonoscopy. Similarly, a separate study conducted in Korea reported cut-off values of 2.26 for NLR and 179.8 for PLR in detecting UC, further supporting the utility of these inflammatory markers in clinical practice [17].

Similar to NLR, PLR is recognized as a novel inflammatory marker. In this study, PLR levels were significantly higher in the UC group compared to the IBS group, consistent with findings in the existing literature. Previous research has indicated that PLR is elevated during the active phase of UC compared to remission, suggesting its potential as a marker for reflecting disease activity and assessing the severity of intestinal inflammation in patients with UC [17,18].

In the current study, ROC curve analysis demonstrated that the combined use of the five indicators – NLR, PLR, CRP, ESR, and Ca^2+^ – yielded superior predictive performance compared to any single marker. Based on these findings, it is recommended that clinical practice incorporate the combined test of these indicators, with a cut-off value of 0.455, to effectively differentiate between UC and IBS. The IBS group was included for the calibration and testing of inflammatory biomarkers, serving as a reference group. Due to ethical considerations at our institution, the IBS group was used as a substitute for healthy controls, not for differential diagnosis with UC.

In areas of mucosal damage in patients with UC, neutrophils play a critical role as the first immune cells to infiltrate sites of inflammation. Previous research has demonstrated that neutrophils contribute to mucosal injury by producing reactive oxygen species with antimicrobial properties and releasing myeloperoxidase and elastase from their granules, which form fibrous networks to combat infection [19]. Additionally, neutrophils regulate the proliferation of invading microorganisms by secreting pro-inflammatory mediators, thereby aiding in the maintenance of tissue homeostasis [20]. However, this activity is accompanied by significant oxidative stress, which is considered as a key mechanism underlying intestinal damage in IBD.

The anti-inflammatory agent infliximab, a tumor necrosis factor inhibitor, has become a cornerstone of IBD immunotherapy, underscoring the potential of targeting oxidative stress as a therapeutic strategy [21]. In UC, inflammatory activity leads to increased apoptosis of lymphocytes in the thymus and spleen, resulting in elevated peripheral blood neutrophil levels and a reduction in lymphocyte counts during the active phase. These changes are reflected in an increased NLR, which serves as an indicator of the imbalance between these cell populations and the intensity of inflammatory activity.

Platelets play a multifaceted role in the body, contributing not only to hemostatic responses and endothelial tissue repair but also to the regulation of inflammatory processes. Upon bacterial activation, platelets release stored cytokines, inflammatory mediators, and antimicrobial peptides, and through direct interaction with immune cells or secretion of mediators, they help coordinate immune responses to inflammation and infection [22].

Although the specific role of platelets in UC remains unclear, several potential mechanisms have been proposed. First, platelets are broadly activated during inflammatory responses, and this activation exacerbates inflammation through the production of ligands via CD40–CD40L interactions [23]. Second, a possible positive feedback mechanism may exist between platelets and neutrophils, facilitated by CD40–CD40L interactions. This is supported by the observation that activated platelets are frequently localized near sites of significant neutrophil infiltration, particularly within ulcerative lesions. Third, previous research suggests that platelets may aggravate intestinal inflammation by inhibiting lymphangiogenesis, a process that is essential for resolving inflammation. This inhibition could contribute to the chronic inflammation characteristic of IBD [24].

These findings suggest that targeting the interactions between platelets and lymphatic vessels may represent a novel therapeutic approach for IBD, offering potential avenues for more effective management of chronic intestinal inflammation.

Calcium, a vital trace element, is primarily stored in bones and teeth as crystalline deposits. In its ionic form, calcium serves as a versatile intracellular messenger, playing a crucial role in numerous physiological processes. According to the 2013 “Expert Consensus on the Clinical Application of Vitamin and Mineral Supplements in the Prevention and Treatment of Inflammatory Bowel Disease,” hypocalcemia in patients with UC results from various factors. These include reduced calcium intake due to anorexia, steatorrhea, decreased intestinal mucosal absorptive surface, vitamin D deficiency, and the use of glucocorticoids. These factors collectively contribute to calcium malabsorption and inadequate dietary intake, significantly increasing the risk of osteoporosis in patients with UC [25].

Given these risks, it is imperative for clinicians to routinely monitor calcium ion levels during both the diagnostic and treatment phases to ensure comprehensive management of UC and its associated complications.

In conclusion, this study identified NLR, PLR, CRP, ESR, and Ca^2+^ as effective, cost-efficient, and safe biomarkers for the prediction and assessment of disease activity in patients with UC. Among these markers, the combination of all five provides the most robust predictive performance. Future research should focus on integrating additional novel serological inflammatory markers to further improve the accuracy of UC assessments.

However, this study has several limitations. First, it included only hospitalized patients in the active phase of the disease and lacked a control group of patients in remission, which may limit the generalizability of the findings. Second, colonoscopy assessments were performed by different physicians, potentially introducing variability in the interpretation of results. Additionally, the single-center, retrospective design may contribute to selection and observational bias.

To address these limitations, future studies should employ multi-center designs with larger sample sizes and long-term follow-up. Such studies should aim to explore simpler, more cost-effective, and safer serological markers for the prediction and monitoring of UC, facilitating improved patient outcomes and disease management.
